# Effect of Intermetallic Compounds on the Thermal and Mechanical Properties of Al–Cu Composite Materials Fabricated by Spark Plasma Sintering

**DOI:** 10.3390/ma12091546

**Published:** 2019-05-10

**Authors:** Kyungju Kim, Dasom Kim, Kwangjae Park, Myunghoon Cho, Seungchan Cho, Hansang Kwon

**Affiliations:** 1 The Industrial Science Technology Research Center, Pukyong National University, 365, Sinseon-ro, Nam-Gu, Busan 48547, Korea; ngm13@ngm.re.kr; 2 Department of R&D, Next Generation Materials Co., Ltd., 365, Sinseon-ro, Nam-Gu, Busan 48547, Korea; ngm15@ngm.re.kr; 3 Department of Hard Magnets Research, The National Institute of Advanced Industrial Science and Technology (AIST), Shimo-Shidami, Moriyama-ku, Nagoya, Aichi 463-8560, Japan; ds-kim@aist.go.jp (D.K.); k.park@aist.go.jp (K.P.); 4 Department of Materials System Engineering, Pukyong National University, 365, Sinseon-ro, Nam-Gu, Busan 48547, Korea; 5 Department of Composites Research, Korea Institute of Materials Science, Changwon-daero, Seongsan-gu, Changwon-si 51508, Gyeongsangnam-do, Korea; sccho@kims.re.kr

**Keywords:** aluminium composite, copper composite, spark plasma sintering, thermal properties, powder metallurgy, intermetallic compound

## Abstract

Aluminium–copper composite materials were successfully fabricated using spark plasma sintering with Al and Cu powders as the raw materials. Al–Cu composite powders were fabricated through a ball milling process, and the effect of the Cu content was investigated. Composite materials composed of Al–20Cu, Al–50Cu, and Al–80Cu (vol.%) were sintered by a spark plasma sintering process, which was carried out at 520 °C and 50 MPa for 5 min. The phase analysis of the composite materials by X-ray diffraction (XRD) and energy-dispersive spectroscopy (EDS) indicated that intermetallic compounds (IC) such as CuAl_2_ and Cu_9_Al_4_ were formed through reactions between Cu and Al during the spark plasma sintering process. The mechanical properties of the composites were analysed using a Vickers hardness tester. The Al–50Cu composite had a hardness of approximately 151 HV, which is higher than that of the other composites. The thermal conductivity of the composite materials was measured by laser flash analysis, and the highest value was obtained for the Al–80Cu composite material. This suggests that the Cu content affects physical properties of the Al–Cu composite material as well as the amount of intermetallic compounds formed in the composite material.

## 1. Introduction

Heat dissipation and the development of lightweight materials are important concerns for the automobile, aerospace, optical material panel, electronic packaging, and semiconductor component industries, among others [1,2,3,4,5,6,7,8]. Given the increasing global enforcement of carbon dioxide emission regulations, these lightweight materials should be eco-friendly and economical, reduce carbon dioxide emissions, and improve the fuel efficiencies of automobiles, shipbuilding, and aviation applications. Aluminium, which has a low density, and copper, which has a high heat dissipation capacity, have attracted attention as suitable materials to satisfy various industrial needs. Recently, the demand for high-functionality materials that are lightweight and have high heat dissipation, low thermal stress, and high strength characteristics has increased to improve the fuel efficiency of transportation and realise miniaturisation of integrated circuits [9,10,11,12,13,14,15,16]. As a result, alloy or composite-related studies have been conducted owing to the demands for various functions in a single material. Low-density aluminium has a relatively lower hardness than other metals; thus, materials such as Fe, Mg, SiC, B, Ti, V, diamond, and carbon nanotubes (CNT) are added as composites to increase the hardness [17,18,19,20,21,22,23,24,25,26,27,28,29,30,31,32,33]. Kwon et al. [34] fabricated Al–CNT composites through a combination of spark plasma sintering and a hot extrusion process. The tensile strength of the Al–CNT composite was 194 MPa, which is twice that of pure bulk Al. The increase in the mechanical characteristics was attributed to the effects of the particular strengthening by CNT and the regularly oriented CNTs achieved through the nanoscale dispersion method. Metal alloys and composite materials such as Al, Cu, Cu–W, Cu–Mo, and Al–SiC have been widely used as heat dissipation materials with high thermal conduction and low thermal expansion coefficient characteristics [35,36,37]. However, cost is an important factor in industrial applications, and the complex manufacturing process and high price of these materials limits their suitability for industrial applications. Recently, interest in Al and Cu composites is increasing, as these composites combine the lightweight properties of Al and the thermal characteristics of Cu. Studies of these composites have been performed using the accumulative roll bonding (ARB) process and the squeeze casting method. Eizadjou et al. [38] used Al 1100 and Cu strips to investigate the mechanical characteristics of the modified structure of a multi-layered Al/Cu composite made with the ARB process. It was reported that as the average thickness of the Cu layer decreased from 100 μm to 7 μm, the strength and hardness increased. However, the thermal characteristics of the Al–Cu composite for use as a heat dissipation material were not investigated. Wu et al. [39] investigated the effects of adding Cu on the thermal characteristics of an Al–Cu/diamond composite manufactured by squeeze casting. It was reported that the thermal conductivity of the Al–3.0 wt% Cu/diamond composite was 330 W·m^−1^·K^−1^ which is 57% higher than that of the Al/diamond composite. Nevertheless, studies on composites with high Al and Cu content are rarely performed because these manufacturing processes have about three times the density difference between Al and Cu and lead to compound formation between the metals.

In this study, the mechanical ball milling and spark plasma sintering (SPS) composite manufacturing processes were used to fabricate a composite material combining the advantages of Al and Cu. The microstructure and form of the composite material were analysed using X-ray diffraction (XRD), scanning electron microscopy (SEM), field-emission SEM (FE-SEM), and energy-dispersive spectroscopy (EDS). The mechanical characteristics of the Al–Cu composite material were measured using a Vickers hardness tester, and the thermal characteristics were measured using laser flash analysis.

## 2. Materials and Methods

The raw powders used in this study were pure Al (99.9%, Metalplayer Co., Ltd., Incheon, Korea) and Cu (99.9%, Metalplayer Co., Ltd.) powders with an average particle size of about 45μm. A mixture of zirconia (diameter of 15 mm) and stainless balls (diameter of 8 mm) in a stainless-steel jar were used for the ball milling process and with the ball mill (SMBL-6, SciLabMix^TM^, Programmable Ball Mill). In all experiments, a specific amount of starting materials were used, giving balls to powder weight ratios of 3:1. Pure Al powders and pure Cu powders were combined as Al–20Cu, Al–50Cu, and Al–80Cu (vol.%) and mixed with 50 ml heptane as a process control agent (PCA) in the stainless-steel jar; the ball milling process was then performed for 24 h at 420 rpm under the ambient atmosphere. The PCA was eliminated by natural evaporation. The composite powders were placed in a graphite mold (diameter of 20 mm), held for 5 min at 520 °C, and sintered using spark plasma sintering equipment (Fuji Electronic Industrial Co., Ltd., SPS-321Lx, Saitama, Japan) with a compacting pressure of 50 MPa. Al–Cu sintered bodies were fabricated with a diameter of 20 mm and a thickness of 5 mm. The density of the composite material was measured with densitometer using the Archimedes method, and the theoretical density was calculated based on the mixture of pure Al and Cu. XRD patterns for the Al–Cu composites were obtained using an X-ray diffractometer (Ultima IV, Rigaku, Tokyo, Japan) with a Cu Kα radiation source (λ = 1.5148 Å, 40 kV, and 40 mA) in the 2-theta range of 20–80° using a linear detector (D/tex ultra, Rigaku).

The microstructures and relative composition of the composite materials were analysed with SEM (VEGA II LSU, TESCAN, Czech Republic), FE-SEM (MIRA 3 LMH In-Beam, TESCAN, Czech Republic), and EDS (HORIBA, EX-400, Kyoto, Japan). Measurements of the area fraction of each component in the Al–Cu composites were performed through digital image analysis using the ImageJ software, which is a version of NIH Image (US National Institutes of Health, http://rsb.info.nih.gov/nih-image). The mechanical properties of the composite materials were determined according to JIS B 7725 and ISO 6507-2 standard using a load of 0.3 kg for 5 s (HM-101 Vickers hardness tester, Mitutoyo Corporation, Kawasaki, Japan); at least five measurements were performed for each sample. The thermal diffusivities and heat capacity of the composites were measured at room temperature with a laser flash apparatus (LFA467, Netzsch, Selb, Germany) according to ISO 22007-4, ISO 18755 and ASTM E 1461 standard. The accuracy of the measuring device is according to the manufacturer ±3% for thermal diffusivity measurements and ±5% for heat capacity measurements. The laser flash method is used to measure thermal diffusivity in a variety of different materials. An energy pulse heats one side of a plane-parallel sample, and the resulting time-dependent temperature increase on the backside due to the energy input is detected. The thermal conductivity, λ, is defined as the ability of a material to transmit heat, and it is measured in watts per square metre of surface area for a temperature gradient of 1 K per unit thickness of 1 m. The thermal diffusivity (α) and heat capacity (Cp) measured by the laser flash method has the following relationship to the thermal conductivity (κ):

λ(T) = α (T) × Cp(T) × ρ(T)(1)

where Cp is the heat capacity, and ρ is the density.

## 3. Results and Discussions

**Figure 1:**
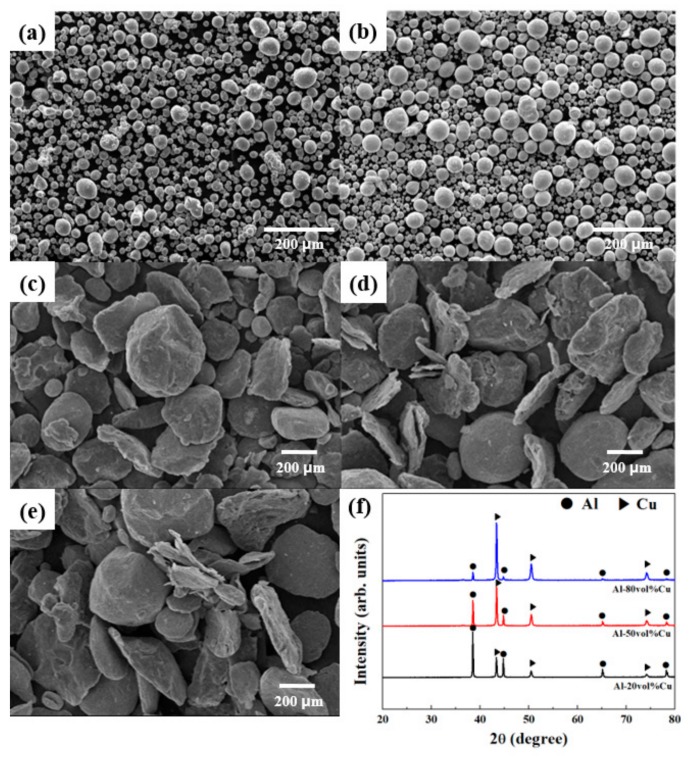
Scanning electron microscopy (SEM) images of (**a**) pure Al, (**b**) pure Cu, (**c**) Al–20Cu, (**d**) Al–50Cu, and (**e**) Al–80Cu powders, and (**f**) X-ray diffraction (XRD) patterns of the composite powders.

Figure 1 shows the morphologies of the pure Al and Cu powders. The pure Al powder exhibits mostly spherical particles, with some ellipse forms, as shown in Figure 1a. As shown in Figure 1b, the pure Cu powder is composed of a dispersion of various sizes of spherical particles. At this point, it was assumed that the pure Al had a natural oxide on the particle surfaces. The morphologies of the Al–20Cu, Al–50Cu, and Al–80Cu composite powders created through the ball milling of pure Al and Cu powders are shown in Figure 1c–e, respectively. In the ball milling process, the powders were milled with plastic deformation as stresses accumulate in the powders through continuous impact with the balls. The Al–Cu composite powders exhibit morphologies containing a mixture of flake and plate-like particles and particles with surface deformation. Ductile particles are easily deformed by the impact energy of the balls, and thus the Al particles with higher ductility than the Cu particles exhibit flake and plate forms. This shows that the impact energy employed during the ball milling under this process condition has enough energy to achieve plastic deformation of the Al particles and deform only the surfaces of the Cu particles. In addition, ductile materials are generally easily aggregated by ball milling, but the Al–Cu composite powders exhibited no aggregate formation and a suitable distribution of Al and Cu. It is desirable for the dispersion in the matrix of the Al–Cu composite material to yield a relatively equal distribution. Figure 1f shows the XRD patterns for the Al–Cu composite powders prior to the SPS process. The XRD pattern results suggest that there were no reactions between the powders during the ball milling process, as the spectra for the Al–20Cu, Al–50Cu, and Al–80Cu composite powders all contained only peaks corresponding to Al and Cu.

**Figure 2:**
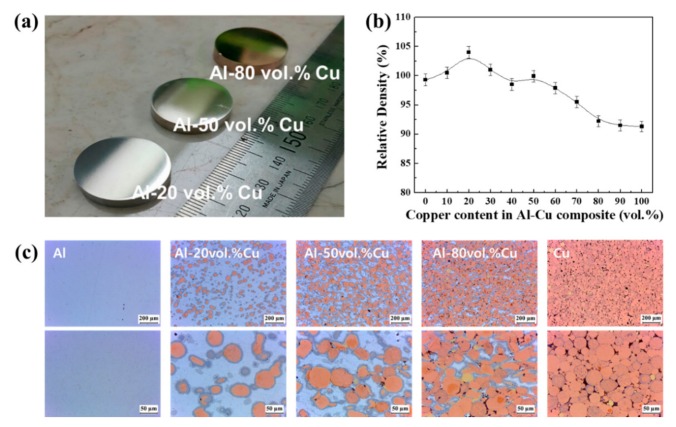
(**a**) Photograph of the Al–Cu composites after spark plasma sintering (SPS), (**b**) evolution of the relative density of composites, and (**c**) cross-sectional light microscopy images.

Figure 2a shows a photograph of the Al–20Cu, Al–50Cu, and Al–80Cu composites after SPS. The Al–Cu composite material in this study was composed of a circular plate having an identical inner structure of a circular carbon mould. As the Cu content in the Al–Cu composite material increases from 20 to 50 and 80 vol.%, the colour changes from light silver to dark copper. Figure 2b shows the relative density of Al–20Cu, Al–50Cu, and Al–80Cu composites after SPS. The theoretical density of the composite materials was calculated based on the Al and Cu mixture composition and the density of Al and Cu. The relative density was calculated by dividing the theoretical density by the measured density (Rd = Ed/Td). Al–20Cu composite exhibits relative densities of about 100% or more and achieved full density during the SPS process in a short period of time. Composites with high relative densities at relatively low sintering temperatures could be successfully obtained through the SPS process. At this point, the Al–Cu composites with a relative density of greater than 100% were considered to no longer have the form of only pure Al and Cu, but rather to contain a different phase. The Al–Cu composites with Cu contents of greater than 50 vol.% exhibited a decrease in the relative density to approximately 90% with increasing Cu content. This indicates that the densification of the composite at relatively low temperature was difficult to achieve for high density owing to the higher melting point of pure Cu than pure Al.

The results indicate that in the Al–20Cu and Al–50Cu composites, Al performs the matrix role because it does have a continuous connection, and Cu functions as a dispersed phase, showing that it is an Al-matrix composite. In contrast, Cu performs the matrix role in the Al–80Cu composite. Furthermore, during the SPS process, it is considered that the Al and Cu reacted to form a different phase than pure Al and Cu at the interface between the Al and Cu. Both the Al–50Cu and Al–80Cu composites formed an identical new phase, which has a pore area corresponding to the relative density. It is suggested that Al–Cu composites can be fabricated by the SPS process without Cu agglomeration and with a uniformly dispersed structure.

**Figure 3:**
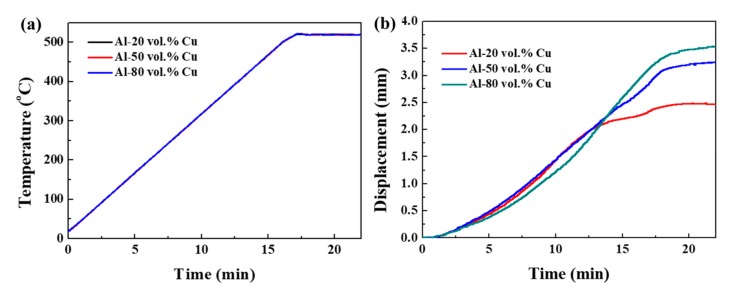
Variation of (**a**) the temperature and (**b**) displacement, as function of holding time SPS.

The displacement profile recorded during the SPS of Al–Cu powders is shown in Figure 3a,b. During the SPS process, the pressure was set to 50 MPa. The temperature was maintained constant at 520 °C for 5 min. The sintering temperature was raised to be similar to the set temperature. As it can be seen during the sintering process, the displacement increases to approximately 3 mm. The displacement was attributed to the particle rearrangement facilitated by the temperature increase. The displacement of Al–Cu composites remains almost constant as the material reaches its maximum density.

**Figure 4:**
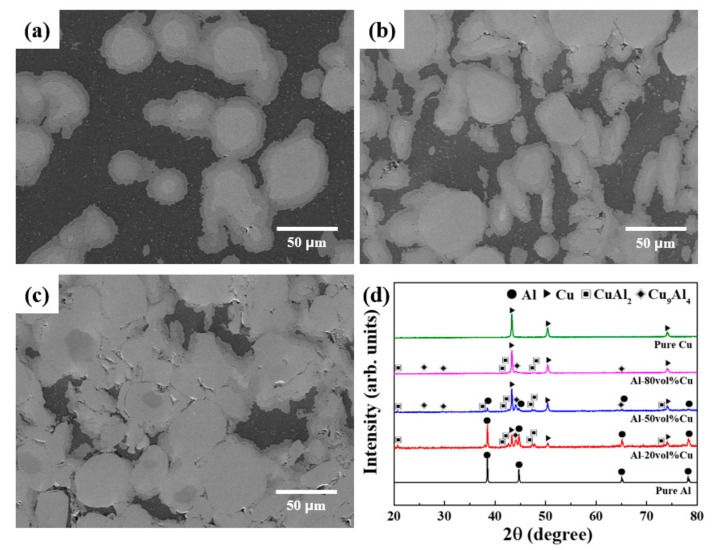
SEM micrographs of (**a**) Al–20Cu, (**b**) Al–50Cu, and (**c**) Al–80Cu composites, and (**d**) XRD patterns of Al–Cu composites.

Figure 4a–c shows SEM micrographs of the Al–20Cu, Al–50Cu, and Al–80Cu composite materials. In Figure 4, the Al–20Cu and Al–50Cu composites fabricated by SPS exhibit fully densified behaviour. SPS is capable of sintering in a short period of time and can provide microstructural control that cannot be expected in conventional sintering processes. In addition, SPS surface treatment can reduce the impurities and oxides present on particle surfaces. Consequently, high-quality and high-density sintered materials can be obtained in a short amount of time. The Al–Cu composites produced by SPS exhibit Al as the darkest phase, Cu as the brightest phase, and two layers between the Al and Cu interface. Figure 4d shows the XRD pattern for the Al–Cu composite powder subjected to SPS. Intermetallic compounds composed of the Al phase and Cu phase as well as CuAl_2_ and Cu_9_Al_4_ phases were detected in the XRD patterns of the Al–20Cu, Al–50Cu, and Al–80Cu composites. In the Al–Cu composite material, the natural air-formed oxide layer on the Al surface was removed by the micro-plasma generated between particles during the SPS process. In addition, the formation of intermetallic compounds is induced by the activation of intermetallic reactions by local high temperatures. The possible chemical reactions in the Cu–Al binary system are as follows:

4Al + 9Cu = Cu_9_Al_4_ (2)

2Al + Cu = CuAl_2_ (3)

Al + Cu = CuAl(4)

According to the standard Gibbs free energy of formation values for chemical reactions (2) to (4), they are all forward reactions, indicating the possibility for formation of intermetallic compounds. The heats of formation for the phases in these chemical reactions are CuAl_2_: −6.1 kJ·mol^−1^, CuAl: −5.1 kJ·mol^−1^, and Cu_9_Al_4_: −4.1 kJ·mol^−1^ [40,41]. The heats of formation for these intermetallic compounds can thus be arranged in order from smallest to largest as CuAl_2_, CuAl, and Cu_9_Al_4_. The CuAl_2_ phase will be formed first, followed by CuAl and Cu_9_Al_4_. However, in the XRD diffraction patterns for the Al–Cu composites subjected to SPS, only the CuAl_2_ and Cu_9_Al_4_ phases were detected; the CuAl phase was not observed. This result occurred because the Cu and Al atoms more readily diffuse into Cu_9_Al_4_ than CuAl, and thus the CuAl phase is not formed. This suggests that kinetic factors are more dominant than thermodynamic factors for the formation of intermetallic compounds. In addition, these results support previous studies that have suggested that kinetic factors are more dominant than thermodynamic factors for the formation of intermetallic compounds. 

**Figure 5:**
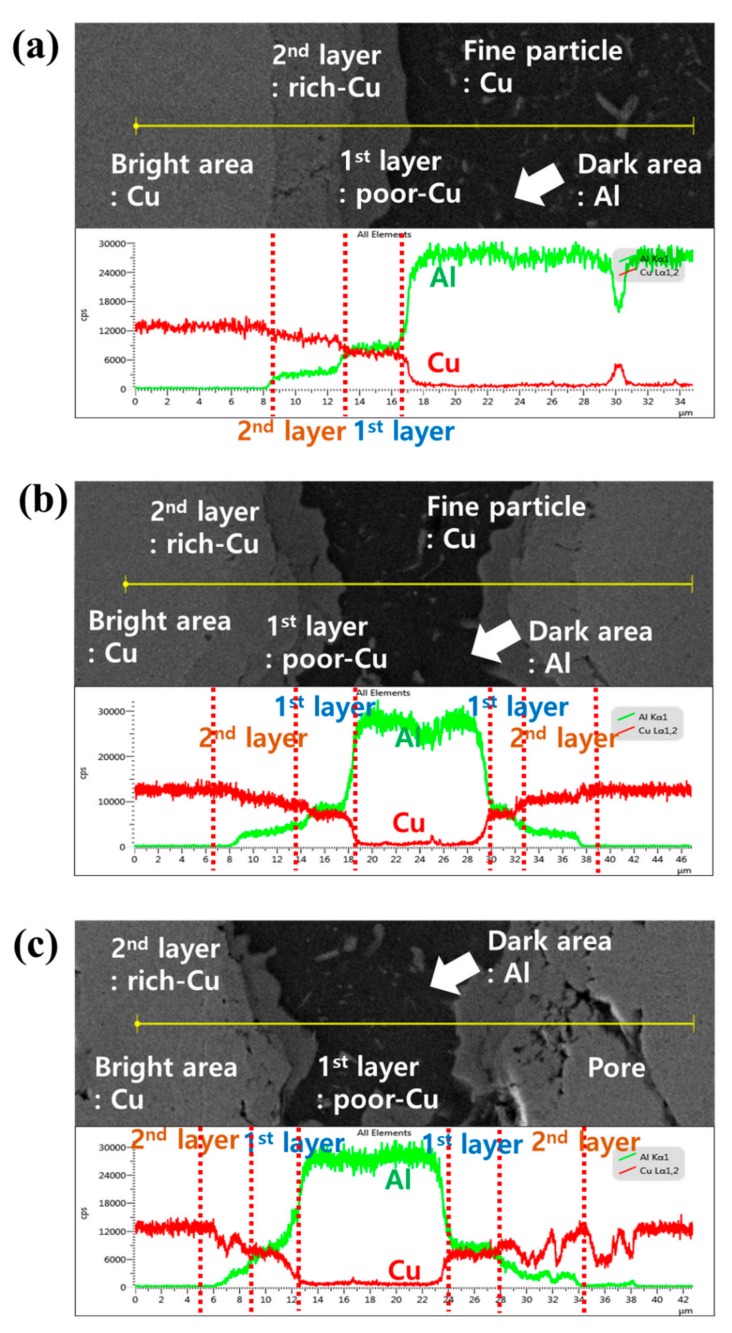
SEM micrograph and energy-dispersive spectroscopy (EDS) line scanning results along the yellow line for (**a**) Al–20Cu, (**b**) Al–50Cu, and (**c**) Al–80Cu.

Figure 5 shows the line mapping images for the Al–20Cu, Al–50Cu, and Al–80Cu composites after SPS. The brightest grey particles confirm the formation of pure Cu phase in the Al–Cu composite material, while the dark area shows the pure Al phase. Of the two IC layers between the Cu and Al phases, the IC layer closer to the Cu is observed to have a higher Cu content than the IC layer closer to the Al. The IC layer that formed closer to the Cu is the Cu-rich Cu_9_Al_4_ phase detected in the XRD pattern in Figure 4d, while the layer closer to the Al is the Al-rich CuAl_2_ phase. The Al–20Cu, Al–50Cu, and Al–80Cu composites exhibited a thicker Cu_9_Al_4_ phase than CuAl_2_ phase. According to the heat of formation for the IC phase, the CuAl_2_ phase is formed first on the interface. However, the Al–20Cu, Al–50Cu, and Al–80Cu composites showed greater growth of the Cu_9_Al_4_ phase than the CuAl_2_ phase. This should be considered a kinetic rather than thermodynamic effect. Xu et al. [39] reported activation energies based on the growth of CuAl_2_ and Cu_9_Al_4_ at Cu–Al interfaces with Cu–Al wire bonds. The activation energy for the growth of the Cu_9_Al_4_ phase is 75.61 kJ·mol^−1^ and that for the CuAl_2_ phase is 60.66 kJ·mol^−1^, which means that the CuAl_2_ phase with a lower activation energy readily participates in the IC phase growth. This suggests that the CuAl_2_ phase is formed first at the Cu–Al interface, and then Cu_9_Al_4_ is formed through the diffusion of Cu atoms into the CuAl_2_ at the CuAl_2_–Cu interface, after which Cu_9_Al_4_ and CuAl_2_ grow simultaneously. At high temperatures, the Cu_9_Al_4_ phase grows more rapidly due to the difference in the diffusion rates of Al atoms and Cu atoms into the formed CuAl_2_ phase, which facilitates the growth of the Cu_9_Al_4_ phase until all of the CuAl_2_ phase is consumed. However, it is considered that two phases coexist because the Al–Cu composites were subjected to the SPS process with a relatively high temperature and short heating period.

**Figure 6:**
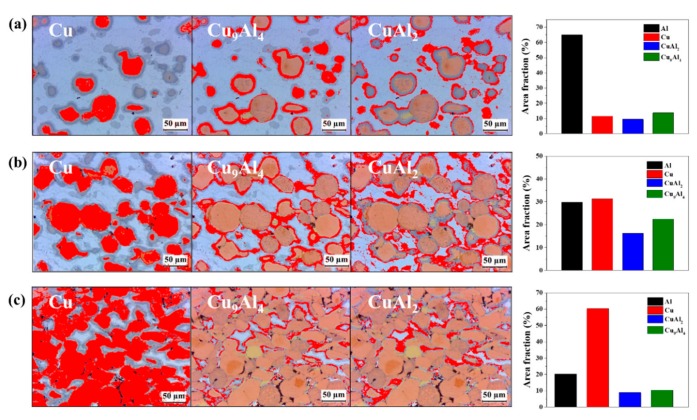
Area fractions of Al, Cu, Cu_9_Al_4_, and CuAl_2_ obtained using image analysis software ImageJ: (**a**) Al–20Cu, (**b**) Al–50Cu, and (**c**) Al–80Cu.

As shown in the XRD diffraction pattern in Figure 4 and the EDS analysis in Figure 5, it was confirmed that not only Cu and Al phases, but also intermetallic compounds of CuAl_2_ and Cu_9_Al_4_ phases, were present in the Al–Cu composites fabricated by SPS. The area analysis for each formed phase was performed using the image analyser tool in the ImageJ software, which measures the area fraction of the phases. The area fractions of the Al–20Cu, Al–50Cu, and Al–80Cu composites were measured using the ImageJ programme. For the light micrographs of Al–Cu composites fabricated by SPS, analysis using the ImageJ analysis toolbox was used to obtain the following area fractions: (i) Al area, (ii) Cu area, (iii) CuAl_2_ area in the intermetallic compounds, and (iv) Cu_9_Al_4_ area in the intermetallic compounds. Figure 6 and Table 1 present the analysis results from the ImageJ programme for a section of the Al–Cu composites after SPS. The area analysis results confirm that the Al and Cu contents of the Al–20Cu, Al–50Cu, and Al–80Cu composites were approximately similar to the desired compositions. The area of the Cu_9_Al_4_ phase in the Al–Cu composites is approximately 1.2 times larger than area of the CuAl_2_ phase. This confirms that the growth of the Cu_9_Al_4_ phase in the SPS process at high temperature is faster. The total IC phase areas in the Al–20Cu, Al–50Cu, and Al–80Cu composites are approximately 23.3%, 38.7%, and 19.3%, respectively; the area of the Al–50Cu composite is the largest. This indicates that the growth of Cu_9_Al_4_ is kinetically faster as the effective contact area between CuAl_2_ and Al or Cu increases.

**Figure 7:**
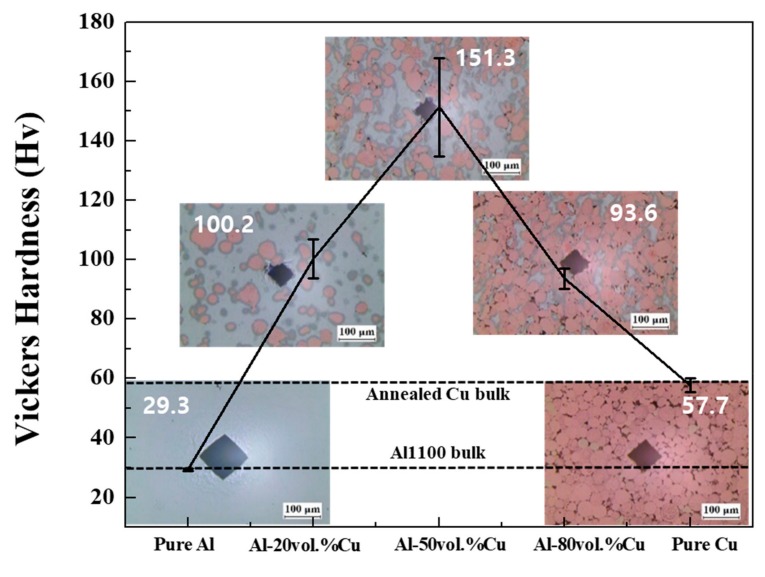
Vickers hardness of the fabricated pure Al, pure Cu, and Al–Cu composites.

Figure 7 shows the Vickers hardness of the Al–Cu composites and pure Al and Cu. The hardness value of a pure Al body fabricated by SPS was similar to the hardness value of Al1100 bulk, while the harness of the Cu sintered body was lower than that of annealed Cu bulk owing to existence of pores [42,43]. However, the Vickers hardness values of the Al–Cu composites were higher than that of pure Al and Cu regardless of the composition. Interestingly, the Vickers hardness of the Al–50Cu composite was about 151.3 HV, which is approximately five times greater than that of the pure Al sintered body and about 2.6 times greater than that of the pure Cu sintered body. It is suggested that this strengthening of the Al–Cu composites was affected by the presence of the ICs. This is mainly because at a higher temperature, Cu and Al will react to form intermetallic compounds, and these intermetallic compounds have a much higher hardness, thus resulting in a significant increase in the hardness at the Cu–Al interface. Under the same sintering condition, Cu and Al form a substitutional solid solution, thus resulting in lattice distortions, which can increase the resistance to dislocation movement. Therefore, plastic deformation becomes difficult, eventually leading to a significant increase in the hardness at the bonding interface. Moreover, the distribution and amount of intermetallic compounds in the Al–Cu composite materials may also be an important factor. As presented in Figure 6 and Table 1, the amount of intermetallic compounds in the Al–50Cu composite was greater than that in the other composites. Thus, the Vickers hardness of the Al–50Cu composite material was the highest because the intermetallic compounds surrounding Cu particles were uniformly dispersed. Therefore, SPS can be considered an effective process for the fabrication of composites of dissimilar materials.

Table 2 lists the thermal conductivities calculated from the thermal diffusivity and heat capacity values for the Al–Cu composites measured with the laser flash method. The pure Al and Cu sintered bodies have values that are similar to the theoretical thermal conductivities based on the mixture composition. However, the thermal conductivity of the Al–Cu composites were lower than the theoretical values and lower than that of the pure Al sintered body. In particular, the Al–50Cu composite with a higher content of intermetallic compounds exhibited a lower thermal conductivity. The interfaces of the Al, Cu, and intermetallic compounds play a critical role in determining both the microstructure and thermal conduction behaviour of the composites. The thermal conductivity of the composites decreased with increasing intermetallic compound content, because the movement of electrons and phonons is disturbed by phase interfaces, and scattering occurs with the formation and the amount of intermetallic compounds. The results show that the intermetallic compounds formed during SPS have a positive effect on the hardness of the Al–Cu composites, but a negative effect on their thermal conductivity. However, investigations using X-ray photoelectron spectroscopy, transmission electron spectroscopy, and on controlling the formation of intermetallic compounds are needed to investigate the enhancement of the material properties, and will be the focus of future studies.

## 4. Conclusions

Al–Cu composite materials were successfully prepared using mechanical ball milling and SPS. The Cu powder was dispersed in Al powder through the ball milling process, and the obtained composite powders were analysed using SEM. It was determined that the ball milling process was suitable for preparing composite powders. ICs were created from reactions between the Al and Cu during SPS, and their presence in the composites was confirmed by the XRD and EDS analysis results. Regardless of the composition, the Al–Cu composite materials exhibited higher Vickers hardness values than pure Al and Cu. The Al–50Cu composite exhibited the highest Vickers hardness of approximately 151 HV. The observed strengthening effects are considered to be related to the formation of intermetallic compounds, which were formed from the reaction between Al and Cu via micro-plasma sparks during the SPS process and detected in the X-ray diffraction and EDS analyses. The Al–50Cu composite with a higher content of intermetallic compounds exhibited lower thermal conductivity. It is suggested that the properties of the Al–Cu composites were affected by the presence of the ICs. Nevertheless, SPS can be considered an effective process for fabricating Al–Cu composite materials.

## Figures and Tables

**Table 1:** Area fraction analysis results for CuAl_2_ and Cu_9_Al_4_ in the Al–Cu composites.

Phase	Partition Fraction (%)		
	Al–20Cu	Al–50Cu	Al–80Cu
**Aluminium**	64.9 (±3.24)	29.8 (±1.49)	20.2 (±1.01)
**Copper**	11.5 (±0.57)	31.3 (±1.56)	60.3 (±3.01)
**CuAl_2_**	9.7 (±0.62)	16.3 (±0.97)	8.9 (±0.45)
**Cu_9_Al_4_**	13.9 (±0.54)	22.6 (±0.96)	10.6 (±0.51)

**Table 2:** Thermal properties and densities of Al, Cu, and Al–Cu composites at room temperature.

Sample	Density			Heat Capacity (J·g^−1^·K^−1^)	Diffusivity		Thermal Conductivity	
	Theoretical Density (g·cm^−3^)	Experimental Density (g·cm^−3^)	Relative Density (%)		Theoretical Diffusivity (mm^2^/s)	Experimental Diffusivity (mm^2^/s)	Theoretical Thermal Conductivity (W·m^−1^·K^−1^)	Experimental Thermal Conductivity (W·m^−1^·K^−1^)
**Pure Al**	2.70	2.680	99.3	0.99	97	81.96	230	219
**Al–20Cu**	3.95	4.111	104.0	0.84	100	46.09	264	158
**Al–50Cu**	5.83	5.826	99.9	0.67	105	33.55	316	130
**Al–80Cu**	7.71	7.110	92.2	0.59	110	45.62	367	191
**Pure Cu**	8.96	8.178	91.3	0.50	113	83.9	401	341
